# HIV-1 Genetic Diversity in Antenatal Cohort, Canada

**DOI:** 10.3201/eid1108.040877

**Published:** 2005-08

**Authors:** Bertine S. Akouamba, Janique Viel, Hugues Charest, Natacha Merindol, Johanne Samson, Normand Lapointe, Bluma G. Brenner, Richard Lalonde, P. Richard Harrigan, Marc Boucher, Hugo Soudeyns

**Affiliations:** * Hôpital Sainte-Justine, Montreal, Quebec, Canada;; † Université de Montréal, Montreal, Quebec, Canada;; ‡ Institut National de Santé Publique du Québec, Sainte-Anne-de-Bellevue, Quebec, Canada;; § Lady Davis Institute for Medical Research, Montreal, Quebec, Canada;; ¶ McGill University Health Center, Montreal, Quebec, Canada;; # British Columbia Centre for Excellence in HIV/AIDS, Vancouver, British Columbia, Canada

**Keywords:** AIDS, HIV-1, viral subtypes, pregnancy

## Abstract

We studied HIV genetic diversity in a cohort of 127 pregnant, HIV-infected women who received prenatal care at Sainte-Justine Hospital in Montreal, Canada, between 1999 and 2003. Clade assignments were derived by phylogenetic analysis of amplified *pol* sequences. Genotyping was successful in 103 of 127 women, 59 (57.3%) of whom were infected with clade B HIV-1, and 44 (42.7%) with nonclade B viruses, including subtypes A, C, D, F, G, and H. Four sequences remained unassigned. Forty-three of 44 women infected with non-clade B viruses were newcomers from sub-Saharan Africa, and subtype identity was consistent with those circulating in their countries of origin. These results highlight the epidemiologic importance of non-B HIV-1 in antenatal populations in a large North American urban center, underscore the influence of population movements on clade intermixing, and identify a group of patients who could be targeted for surveillance and drug therapy followup.

## Abstract 2

Non-B HIV-1 was consistent with patients’ area of origin.

HIV-1 exhibits considerable genetic diversity resulting from the high mutation rate of reverse transcriptase, high viral turnover, viral genomic recombination, and immune and therapeutic selection pressures (1–3). This diversity is a challenge for viral load determination, drug resistance testing, and AIDS vaccine development (1,4–6). Three phylogenetic groups of HIV-1, main (M), outlier (O), non-M, non-O (N), are recognized (2,7). Most HIV-1 infections are caused by group M viruses that comprise 9 clades (A–D, F–H, J, and K) and >13 intersubtype recombinants known as circulating recombinant forms (CRFs) (8). Clade B is most common in North America, Europe, and Australia. However, in the last decade, prevalence of infection with nonclade B viruses has increased in France, Belgium, Spain, and Switzerland (9–12), in large part after migration from or international travel into HIV-endemic areas (13). Nonclade B viruses also circulate in Cuba (14) and the United States (15,16). We measured HIV-1 subtype diversity in a multiethnic cohort of pregnant, HIV-infected women to determine whether nonclade B HIV-1 is emerging in Canada after population movement, and whether antenatal cohorts are suitable sentinel sites to monitor the introduction of nonclade B viruses into Canada.

## Patients and Methods

### Patients

One hundred twenty-seven HIV-infected women receiving prenatal care at Centre Maternel et Infantile sur le SIDA, Sainte-Justine Hospital, Montreal, from October 1999 to September 2003 were included in the study. Inclusion criteria were 1) age ≥18 years, 2) a request for prenatal care, 3) positive HIV-1 serologic results, and 4) informed consent. Standardized clinical followup, including antiretroviral (ARV) prophylaxis and treatment, was provided to all women and their children. This cohort study was conducted according to the guidelines of the Ethics Review Board of Sainte-Justine Hospital.

### Clinical Parameters

HIV-1 serologic status was determined by using the AxSYM HIV 1/2 gO method (Abbott Diagnostics, Wiesbaden, Germany) and confirmed by Western blot. HIV-1 viral load was measured by using the Versant HIV-1 RNA 3.0 assay (bDNA, Bayer, Pittsburgh, PA, USA). CD4+ T-cell counts were measured by flow cytometry. Standardized data collection assessed sociodemographic variables and previous and current ARV treatment. Numeric variables were compared by using the Kruskal-Wallis test. Categoric variables were examined by using the Fisher exact test (SPSS version 11.0, SPSS, Inc., Chicago, IL, USA).

### HIV-1 Genotyping

In cases in which viral load was >1,000 RNA copies/mL plasma, HIV-1 genotyping was performed by using a protocol (Virco BVBA, Mechelen, Belgium) based on sequencing of a 1,497-bp fragment of the HIV-1 *pol* gene (position 2253-3749). In cases in which viral load was <1,000 copies/mL, viral RNA was extracted from plasma, and a 524-bp *pol* segment (position 2597–3120) was amplified by using primers 3069R (5´-GGA TGG CCC AAA GGT TAA ACA-3´) and 3591F (5´-ATC CTA CAT ACA AAT CAT CCA T-3´) and the QIAamp 1-step reverse transcription–polymerase chain reaction (RT-PCR) method (Qiagen, Mississauga, Ontario, Canada). PCR conditions were 40 cycles consisting of 94°C for 30 s, 53°C for 1 min, and 72°C for 1 min, followed by extension at 72°C for 10 min. Amplicons were cloned into pPCR-Script (Stratagene, La Jolla, CA, USA) and sequenced by using dye terminator chemistry (Beckman-Coulter, Palo Alto, CA, USA).

Sequences were aligned with references (2001) representing different HIV-1 subtypes (http://hiv-web.lanl.gov) (8) by using Clustal X version 1.81 (17). Kimura 2-parameter distance matrices were assembled (transition/transversion ratio of 2) (18,19). Phylogenetic reconstructions were built according to the neighbor-joining method, and 1,000 bootstrap resamplings were performed to assess tree topology (MEGA version 2.1) (20). Clade assessment was based on reliable grouping (>80% bootstrap) with reference sequences (8). RIP version 1.9 (http://www.hiv.lanl.gov/content/hiv-db/RIPPER/rip_test.html) (21) was used to examine potential intersubtype recombinants, with gap stripping on, a window size of 200 characters, and a significance threshold of 90%.

## Results

One hundred twenty-seven women 18.3–42.6 years of age (median 30.9, interquartile range [IQR] 7.2) were included in the study: 40 (31.5%) from North and Central America, 35 (27.6%) from the Caribbean, 1 (0.8%) from Asia, and 51 (40.2%) from sub-Saharan Africa. Median HIV-1 viral load at the time of inclusion in the study was 3.24 log RNA copies/mL of plasma (IQR 1.97) and median CD4+ cell count was 403 cells/μL (IQR 248). Of the 127 patients, 66 (52.0%) had not received ARV therapy before study inclusion, 8 (6.3%) had interrupted therapy, and 53 (41.7%) were treated with a regimen consisting of 1 (n = 2), 2 (n = 9), 3 (n = 39) or 4 (n = 3) ARV drugs.

The HIV-1 *pol* gene was successfully amplified and sequenced in 103 (81.1%) of 127 patients, a rate comparable with findings of other studies (22). Seventy-three results were obtained with the Virco procedure, and 30 were obtained with an alternative RT-PCR method. Unsuccessful amplification was associated with low viral load: patients with a viremia level of <500 copies/mL accounted for 23 (95.8%) of 24 in whom gene amplification was unsuccessful, in comparison with 27 (26.2%) of 103 in the rest of the study group (p<0.0004, Fisher exact test). This is consistent with the finding that a larger proportion of patients with unsuccessful gene amplification were treated with ARV therapy at the time of inclusion in the study (75.0% versus 34.0%, p<0.0004, Fisher exact test). Despite this limitation, sequence information was obtained in more than half of patients with a viremia level of <500 copies/mL (27/50), and in one third of patients with a viremia level of <50 copies/mL (8/24).

Phylogenetic analysis based on a 524-bp *pol* fragment (position 2597–3120) was used to identify the HIV-1 clade. In all cases, grouping based on the 524-bp fragment was consistent with that obtained when all available 1,497-bp sequences were analyzed separately (data not shown). In aggregate analysis, sequences derived from 59 (57.3%) of 103 patients formed a well-defined cluster with clade B reference sequences (Figure, left panel and data not shown). Of these 59 patients, 27 (45.8%) were of Canadian origin, 27 (45.8%) were from Haiti, 2 (3.4%) from Mexico, 1 (1.7%) from Jamaica, 1 (1.7%) from the Dominican Republic, and 1 (1.7%) from the United States. Phylogenetic overlap between these sequences was considerable, and bootstrap support for clustering based on country of origin was <50% (Figure, left panel).

**Figure:**
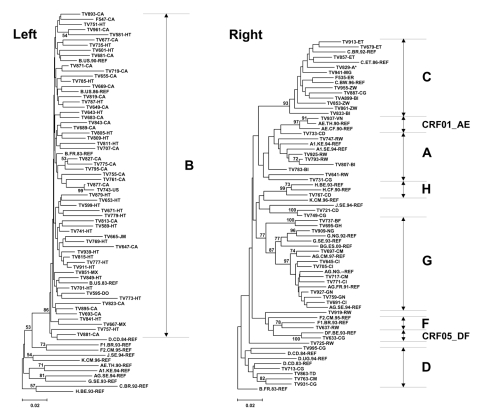
Phylogenetic analysis of *pol* sequences derived from pregnant women infected with HIV-1. Trees were constructed by using the neighbor-joining method as described in Patients and Methods. A transition/transversion ratio of 2 was used and 1,000 bootstrap resamplings were performed. Left panel: Subgrouping with clade B HIV-1. Right panel: Grouping with non-B HIV. Reference sequences (REF) were obtained from the Los Alamos National Laboratory database (2001) (8). The scale bar represents 0.02 nucleotide substitutions per site. Letter codes indicate country of origin. All nucleotide sequence information was submitted to GenBank (accession no. DQ059647–DQ059749). CRF, circulating recombinant form.

In addition, 44 (42.7%) of 103 patients were infected with nonclade B viruses. Nine (20.5%) of the amplified sequences were similar to reference sequences from clade A, including CRF01-AE. Within this cluster, independent grouping of sequences derived from patients TV641, TV731, and TV783 was only supported by low bootstrap values (Figure, right panel). Sequences from 12 patients (27.3%) clustered alongside clade C references (93% bootstrap), with TV833 the distal taxon. Five (11.4%) grouped with clade D. Two (4.55%) grouped with clades F1 and F2, with TV633 closest to the CRF05-DF reference. One sequence (2.27%) grouped with clade H (99% bootstrap), and 11 (25.0%) with clade G. Among these, 8 sequences formed a well-supported CRF02-AG subcluster (97% bootstrap), while TV909 grouped closest to clade G reference (96% bootstrap). TV737 and TV695 formed a distinct G clade subcluster (100% bootstrap) (Figure, right panel). The 938-nucleotide (nt) fragments of the envelope (*env*) gene V1-V3 region were amplified, sequenced, and analyzed in samples from patients TV737 and TV695. These segments clustered closely with one another (96% bootstrap) but loosely with clade G references (41% bootstrap), which confirmed that these 2 isolates fall outside of the subtype G crown group (data not shown). Finally, TV721 and TV749 clustered loosely with the J reference (61% bootstrap), while TV725 and TV919 grouped outside major clades, although all belonged to the M group (100% bootstrap) as determined by phylogenetic analysis using group N, O, U, and SIVcpz alignments (8) (not shown).

In patients in whom the 1,497-nt sequences were available, the potential intersubtype mosaic nature of viruses with uncertain clade assignment was examined using RIP (21). This analysis indicated that TV731 and TV783 had significant homology with the A1 + A2 consensus, TV833 was homologous to the clade C reference, and TV737 and TV909 closely resembled the clade G consensus (>90% confidence), which confirmed initial assessments. The recombinant nature of TV633 was also supported, with significant homology to clades D and F (putative crossover at position 2795–2796), while TV695 showed highest resemblance to clade G in its 5´-terminal portion and clade C at the 3´ end (>90% confidence), with a potential breakpoint at position 3169–3170. In addition, TV721, TV725, TV749, and TV919 did not show significant homology with any of the sequences in the reference alignment, which prevented assessment of their putative intersubtype nature and their assignment to existing M group clades (Figure, right panel and data not shown). TV721 and TV749 were compared with HIV sequences in GenBank using BLAST (http://www.ncbi.nlm.nih.gov/BLAST/). The closest homology to TV721 was isolate A2-225.692 from Uganda (23), with 92% identity over a 522-nt segment. The closest homology to TV749 was isolate 97CM.MP806 from Cameroon (24), with 89% identity over an 884-nt segment. When the 938-nt segments of the *env* gene V1-V3 region were amplified and sequenced, TV721 and TV749 clustered closely with one another (100% bootstrap) and with clade G and J references (92% bootstrap) (data not shown). This finding suggests that TV721 and TV749 represent either complex mosaic recombinants or a new subtype of the HIV-1 M group.

In all but 1 patient (43 [97.7%] of 44), those infected with nonclade B viruses were newcomers from Africa, including 34 (77.3%) asylum seekers. Nine patients originated from West Africa: Côte d'Ivoire (n = 4), Burkina Faso (n = 1), Guinea (n = 2), Ghana (n = 1), and Nigeria (n = 1). Twenty-five originated from central Africa: Congo (n = 7), Democratic Republic of Congo (n = 3), Rwanda (n = 7), Burundi (n = 4), Cameroon (n = 3), and Chad (n = 1). Four originated from East Africa: Ethiopia (n = 3) and Eritrea (n = 1). Four originated from southern Africa: Zimbabwe (n = 3) and Madagascar (n = 1). One patient declined to specify her country of origin. Geographic clustering was observed on the cladogram, with West African sequences grouping among clade G, and East and southern African sequences grouping with clade C. The highest HIV-1 genetic diversity was observed in patients from central Africa (Figure, right panel), as previously reported (25).

Median viral load and CD4+ cell count at the time of inclusion in the study were not significantly different in patients infected with clade B virus versus those infected with nonclade B virus, although more patients infected with clade B virus received ARV therapy. In patients not treated, median CD4+ cell count was 91 cells/μL lower in those infected with nonclade B virus, which suggests more advanced disease (Table). Comparison of duration of infection between subgroups was not possible.

**Table:** Viral and immune parameters in study participants*

	Overall					Treatment naive	
	Median (IQR) viral load (log copies/mL)†	% viral load <2.7 (n)‡	% viral load <1.7 (n)‡	Median (IQR) CD4+ count (cells/μL)†	% receiving ARV therapy (n)‡	Median (IQR) viral load (log copies/mL)†	Median (IQR) CD4+ cell count in treatment-naive patients (cells/μL)†
HIV-1 B clade	3.61 (1.71)	28.8 (17)	11.9 (7)	360 (285)	44.1 (26)	3.95 (1.38)	418 (278)
HIV-1 non-B clade	3.52 (1.39)	22.7 (10)	2.27 (1)	351 (220)	20.5 (9)	3.53 (0.94)	327 (208)
p value	0.927	0.508	0.316	0.476	0.0102§	0.143	0.107

*HIV-1 viral load and CD4+ cell counts were measured as described in Patients and Methods. Significance of differences between groups was tested by Kruskal-Wallis test or Fisher exact test. Analysis was carried out on the whole study group (N = 103) or restricted to those who did not receive treatment (n = 61). IQR, interquartile range; ARV, antiretroviral. †Kruskal-Wallis test. ‡Fisher exact test. §Statistically significant (p<0.05) by directional test.

## Discussion

HIV-1 clade diversity was characterized among a cohort of HIV-infected women receiving prenatal care in a tertiary care hospital serving a cosmopolitan population. Results indicate that 59 (57.3%) of 103 patients in whom genotyping was successful were infected with clade B HIV-1. This finding is compatible with the wide circulation of clade B in North and Central America and the Caribbean, from which 40 (31.5%) and 35 (27.6%), respectively, of the 127 patients in our cohort originated, and the relatively high prevalence of HIV-1 infection among patients from Haiti in the Montreal area (1,26). Additionally, 42.7% of patients in whom genotyping was successful were infected with nonclade B viruses, a proportion much greater than the rate reported in 312 HIV-infected US blood donors (2%) (16) and in a recent Canadian public health surveillance report (8.9%) (27). To our knowledge, this is the highest prevalence of non-B HIV infection reported in any North American study group, including US military personnel (16,28,29). Sequences were identified that belonged to every clade of the HIV-1 M group except J and K. This level of genetic diversity was not previously reported in a North American study group, with the exception of the Centers for Disease Control and Prevention surveillance registry (22), and is as extensive as that observed in Cuba (14). Four of the *pol* segments obtained clustered ambiguously among reference sequences, which suggests that they represent either novel HIV-1 M group clades or complex recombinants. However, additional characterization, including full-genome sequencing, would be required to settle this issue. Based on our results, infection with multiple HIV-1 subtypes cannot be reliably assessed.

A total of 97.7% of non-clade B viruses were found in African women and, in all cases, clade identity was consistent with variants circulating in the patient's area of origin (1). No significant difference was found between the proportions of African women in patients with unsuccessful amplification (8 [33.3%] of 24) versus those in whom amplification was successful (43 [41.7%] of 103, p = 0.496, Fisher exact test), which is indicative of no selection bias. Recent armed conflicts in the African subcontinent have led to an influx into Canada of newcomers from HIV-endemic areas (30,31). Among our study group, dates of arrival into Canada of patients infected with nonclade B HIV-1 correspond with the migration of refugees after the Rwandan genocide and the civil war in the former Republic of Zaire and neighboring Congo (data not shown) (30,31). Nonclade B viruses have spread in Europe and Cuba as a consequence of international travel and immigration from Africa (9–14). Our study demonstrates that multiple HIV-1 clades are being introduced under similar circumstances in a large, North American urban center. From a public health standpoint, antenatal cohorts could represent an important sentinel site to monitor the influx of novel HIV-1 variants in industrialized countries.
